# Food, Feminist Rhetorical Studies, and Conservative Women: The Case of Elizabeth David

**DOI:** 10.1080/07350198.2022.2077035

**Published:** 2022-07-13

**Authors:** Richard Vytniorgu

**Affiliations:** University of Exeter

## Abstract

This article argues for the importance of British food writer Elizabeth David (1913-1992) in questioning the centrality of power in feminist rhetorical studies and thereby furthering our capacity to understand the diversity of conservative women and their rhetorical projects. The article analyzes David’s pathos in her landmark volume of gastronomical essays, *An Omelette and a Glass of Wine* (1986), and shows how this rhetoric develops a conservative “political culture” which privileges human motivations within food cultures that move beyond the negotiation of power.

Elizabeth David (1913-1992) is arguably Britain’s most significant food writer since the Second World War.^1^ Scholars have primarily examined her literary evocations of Mediterranean cuisine in the gloomy post-war years, when rationing dominated British life until meat rationing ended in 1954.^2^ But David has never been read as a rhetor, still less for her ability to speak to feminist rhetorical studies and its increasing engagement with women’s food writing. This article argues that David’s journalism of the post-war years, culminating in her 1986 edited volume of essays, *An Omelette and a Glass of Wine*, develops a conservative rhetoric that is able to bring feminist rhetorical analyses of conservative women into fruitful dialogue with those of food.

I argue that David’s rhetoric is of interest because it questions the centrality of power in feminist rhetorical studies which, I suggest, currently obscures the range and potential of conservatism – especially in Britain – to showcase human motivations other than power. Rather than focusing on David’s negotiation of the power structures available to her as a conservative woman, I examine how her engagement with food and conservative attitudes intersects a conservative “political culture” that diversifies our understanding of women’s conservatism and its rhetorical range, particularly in post-war Britain. Not primarily concentrating on negotiating power, David’s rhetoric uses pathos to emphasize the importance of affective bonds between past, present, and future, and how these bonds generate and conserve tradition which is threatened by modernizing impulses, particularly in relation to food.

The article begins by reviewing the way in which feminist rhetorical studies and work on food have engaged conservative women, and questions whether the most important way to engage conservative women in feminist rhetorical studies is always through understanding how they negotiated power. It then outlines some of the central themes of British—as opposed to American—conservatism that animate David’s own rhetoric. After a brief biographical comment for readers unfamiliar with David and the kind of food writing she produced—gastronomy rather than recipe books—I proceed with a textual analysis of David’s pathos in *An Omelette and a Glass of Wine* and its contribution to a conservative political culture in which power is but one of a number of important human motivations.

## Questioning the Centrality of Power in Feminist Rhetorical Studies

It is now a familiar claim that feminist rhetorical studies wishes to expand the range of women rhetors studied as part of a more comprehensive analysis of power, or “the ways in which women maneuver amid systems of power” (C. Hogg 22).^3^ Such an expansion should, therefore, include conservative women. In *Walking and Talking: Feminist Rhetorical Practices*, Jacqueline Jones Royster and Gesa E. Kirsch ask the important question of how feminist rhetoricians represent women rhetors who do not readily share their feminist outlook on life (22). Charlotte Hogg has added: “[W]e should also look toward women who may not seek to empower themselves or others yet hold rhetorical sway” (397).

But as Hogg notes, what often happens when we explore women rhetors who do not seem to “forward a feminist agenda” is that “our conclusions still tend toward analyzing how their rhetorical acts—intentionally, overtly, subtly—find them doing just that” (C. Hogg 397). Rather than trying to locate a feminist agenda among women rhetors who do not readily fit such a profile, Hogg asks that, to echo Royster and Kirsch, we model an “ethics of hope and care” (398). We need to care about conservative women as part of an overall feminist project of “analyzing the operations of difference and the workings of power” (Scott 70).

Among U.S.-focused scholarship, in recent years feminist critics have paid attention to the conservatism of women’s religious rhetoric and women’s organizations in the late twentieth century, especially concerning speech and sexual morality—themes which clearly scrutinize systems of power.^4^ In the UK, where I write from and where the study of rhetoric has not been as prolific as in the U.S., a body of work outside rhetoric has arisen which seeks to analyze the contributions of conservative women to party-specific activity.^5^ An exception to this trend is Alison Light’s ground-breaking *Forever England: Femininity, Literature and Conservatism Between the Wars*, which identifies a canon of middlebrow fiction written by women in the interwar years and contributes to what political historians such as Lawrence Black are now calling “political culture”—a mode of politics separated from the histories of political parties and organizations and that reach into everyday spheres of life, including the home and, crucial to my purposes here, the kitchen (Black 3).^6^

While these interdisciplinary works have undoubtedly opened up germane lines of inquiry and have begun to address a blind spot in feminism more broadly, the logic nevertheless remains that if women rhetors are to be studied who do not themselves directly or indirectly advance the cause of dismantling patriarchy, then they must at least hold “rhetorical sway” or power to be worthy of analysis and critique, positioned as “women complicit in the patriarchal structures feminism works against” (C. Hogg 398). The hidden (and mistaken) assumptions here are that: (1) conservative women such as David are of a piece with one another, stifling heterogeneity among them; (2) conservative women are either unenlightened and in need of empowerment, or, (3) they unconsciously hold power because they do not try to dismantle patriarchy. Hogg’s response is to focus on an *interplay* between conservative and feminist women with a simultaneous *inquiry* into “the ways power is negotiated and reinforced by those who have it or subscribe to the ideologies of the dominant culture” (C. Hogg 398-99). But there are still two problems with this approach, which this article addresses through reading David’s work.

Firstly, while Hogg dismisses feminist rhetoricians’ reliance on binaries (feminist vs. antifeminist), she effectively erects another by arguing that we embrace subjects whose views either “inspire” or “dismay” us, thereby setting an unhelpful script for appropriate scholarly response that only superficially appears to meet the requirement that feminist rhetoricians engage with conservative women such as David without trying to locate a hidden feminist agenda in their work (399). Secondly, while feminist rhetorical studies both practices and theorizes its own first-person plural—witness the copious use of “we” and “us” in its scholarship—it presumes, perhaps understandably, that this first-person plural is decidedly feminist, and that a single unifying theme for feminist rhetorical studies should be an analysis of how “women maneuver amid systems of power.” Combine this with the field’s tendency to encourage scholars to declare their own subject position—“writing oneself into the story being staged thus becomes a way of writing oneself into history,” to echo Joan Wallach Scott—and there results a pressure to approach conservative women in ways that (1) maintain power as the key interpretive lens in analyzing conservative women and (2) that presumes the correct scholarly stance towards it through talk of “dismay” and an agential first-person plural that writes one largely into a feminist—and hence oppositional—side of a divide among women (51).

My response is to show how feminist rhetorical studies can engage with conservative women rhetors in less prescriptive ways by de-centering the interpretive lens of power. By treating conservatism here as a broader political culture that reaches into the realm of food, I show how conservative food rhetoric—especially in Britain—involves much more than maneuvering amid systems of power associated with more American forms of women’s conservative rhetoric, concerned with abortion, pornography, and the traditional family.

Meanwhile, feminist food rhetoric itself has yet to witness an explicit engagement with conservative women. Melissa A. Goldthwaite’s recent edited volume, *Food, Feminisms, Rhetorics*, largely bypasses Royster’s and Kirsch’s challenge to engage with women rhetors who do not obviously share a contemporary feminist agenda. Abby Dubisar’s essay, for instance, stresses the importance of finding out how women rhetors can “be both engaging and subversive” (60), while Abby Wilkerson has analyzed how feminist food rhetoric is “generating resistance to entrenched power” (129). *Food, Feminisms, Rhetorics* would appear, by and large, to agree with Arlene Voski Avakian and Barbara Haber in prioritizing work that shows “how in their food practices women resist oppression through racism, colonialism, and globalization” (viii). Introducing Elizabeth David into the discussion creates an intersection between feminist food studies and wider feminist rhetorical engagement with conservative women, while also opening the geographical focus to include British women who tap into different philosophical and cultural sources that transform how we understand conservatism as a political culture, concerned with power, yes, but with other things as well that often take center stage.

## The Political Culture of Post-War British Conservatism

Immediately after the Second World War was not a good time to be a Conservative in Britain. In 1945 the Labour Party swept to victory and thereby instigated a period in the wilderness for the Conservatives, who did not return to power until 1951—exactly when David’s career as a writer began to take off. But while political historians have been busy re-evaluating the so-called “inevitability” of the Conservative defeat in 1945, other historians have argued for a more diffusive understanding of conservatism, with a small “c” (Kowol 474).^7^ How did conservative attitudes, perspectives, and ideas develop after the Second World War? Which lines of thought did they attach themselves to in British conservative thinking of the past?

In his *Redefining British Politics: Culture, Consumerism and Participation, 1954-70*, Lawrence Black has argued for the importance of political cultures in shifting attention away from party-specific activity. This means evaluating “politics in its wider social setting, in which as a minority or occasional interest and identity, politics could bear a certain ‘otherness,’ much as ethnicity or social class might. This suggests that political culture might not be very political, measured in conventional terms” (3). In fact, British conservatism is ideal for studying in terms of a broader political culture, given its historic resistance to systematization and its insistence that it is non-ideological, simply a set of attitudes (and possibly behaviors), more than a coherent philosophy.

Applied to the period in which Elizabeth David began her journalism, in the late 1940s, there are a few key elements to bear in mind. The sheer length of rationing, impacting people’s lives for almost a decade after the war ended, meant that discussion about food remained tilted towards health, nutrition, and making ends meet. During the late-1950s and 1960s, as living conditions improved, attention oriented more to quality of life issues. As Black explains: “Politics [in Britain] was increasingly about rights, tastes, culture, morality, environmental, post-industrial, even anti-materialist, desires and self-expression and less about needs” (8). By the 1970s, a coherent and often connected network of women food writers was persuading middle-class readers about the need to conserve food traditions native to England that were at risk of being lost amidst the smorgasbord of ascendant lifestyles to be had.

In this sense, food writers such as Elizabeth David, as well as Jane Grigson (1928-1990) and Elisabeth Ayrton (1915-1991), were forming a political culture in which their conservative rhetoric was focused on recording and preserving food cultures at risk of being lost. Titles such as David’s *Spices, Salt and Aromatics in the English Kitchen* (1970) and *English Bread and Yeast Cookery* (1977), Grigson’s *English Food* (1974), and Ayrton’s *The Cookery of England* (1974) and *English Provincial Cooking* (1980) self-consciously embedded themselves in a history of women’s small-“c” conservative food writing in Britain. As Grigson explains:

One thing to note is that the great English cookery writers from Hannah Glasse to Elizabeth David have always been women, in contrast to the French tradition of cookery writing by male chefs. Our classical tradition has been domestic, with the domestic virtues of enjoyment and generosity […] We need to renew and develop the old [pre-industrial] tradition of Hannah Glasse [1708-1770], Elizabeth Raffald [1733-1781], Maria Rundell [1745-1828] and Eliza Acton [1799-1859] as far as we can in our changed circumstances. (3)

While David, Grigson, or Ayrton cannot be said to have practised an *explicitly* conservative rhetoric, largely because none of them self-identified as conservative or as engaging in a conservative project, their rhetoric, and David’s in particular, can be classified as conservative due to the ends to which such rhetoric was put, developing a specific political culture of food.^8^ As a rhetor, for David the key aim was to communicate a matrix of food cultures by invoking the importance of a living tradition capable of maintaining precious knowledge which was at risk of being lost in a changing world. And, far from being impractically nostalgic—a frequent accusation levelled against conservatives—David’s project was meticulously historical, especially from the 1960s onwards, when she scoured the country in search of original archival material for her work. Here, David’s perspective connects to key British conservative thinkers of the past.

One of these—Edmund Burke—is especially foundational and two of his ideas have been at the crux of British conservative political culture ever since: anti-rationalism (or non-rationalism), and respect for tradition. For Burke, “We are afraid to put men to live and trade on his own private stock of reason; because we suspect that this stock in each man is small, and that the individuals would do better to avail themselves of the general bank and capital of nations and ages” (500). Burke’s suspicion of the rational planning that caused the French Revolution, later taken up by the British philosopher Michael Oakeshott, is the corollary of his interest in, and respect for, history and tradition. Conservative thought in Britain has often been defensive of the particularity of the past which has made conservatives skeptical of universals and, therefore, any “plan” that erects itself as able to transform the conditions of people who differ so much from one another in practice. Instead, for Burke it is crucial to recognize that good things are built slowly, by imperfect people in delicate cultures, and that, often in the name of planning and idealism, such good things are torn down more easily than they are built up. “Tradition” is therefore as much a process as it is a product: it is the means, often faltering, by which knowledge is preserved, not necessarily for its own sake, but for how it advances human happiness in distinct settings.

This mode of conservatism, and the rhetoric which serves its end, is somewhat different from the kind typically discussed in American contexts, as Ronnee Schreiber and others have so carefully explored. Granted, Britain also had people like Mary Whitehouse and Enoch Powell—more obviously conservative rhetoricians who, placing power as a central idea, advanced ideas about sexual morality and racial purity which have since, by and large, been superseded in Britain by more accepting attitudes.^9^ What is more fruitful today, however, is the exploration of an alternative, less obvious conservativism and the rhetoric which served its end. It is a conservatism in which affect, imagination, and reverence (but not subservience) take center stage, existing in Grigson’s non-oppressive feminine arena of “the domestic virtues of quiet enjoyment and generosity” (3).

Indeed, it has been customary among British conservatives to recognize that, contrary to the ideas of Nietzsche and later Foucault, the negotiation of power is not the only or most significant motive steering human behavior, and that a true study of political culture from a conservative perspective must take into account a broader range of dynamics that animate culture and, at any given moment, may be more relevant than power.^10^ As the forerunner to Grigson, Ayrton, and others who studied food as a key facet of British culture, David is key to understanding how and why this expression of conservative political culture, rooted in food traditions, flourished in the post-war years.

## Who was Elizabeth David?

For those unfamiliar with David, a short biographical comment is required.^11^ Elizabeth David (née Gwynne) was born into an aristocratic family in rural Sussex with three Conservative Members of Parliament as family members. Accustomed to servants, David did not learn to cook until the 1930s when she rebelled against her upbringing and pursued an unsuccessful acting career in Oxford and London, and work as a shop assistant at Worth’s—a high-end department store. During this time, she moved in and out of apartments with her working-class, married lover who was also pursuing a career in acting. In 1939, David finally unmoored herself from her family and sailed round the Mediterranean with her lover and learned how to cook Mediterranean dishes with ingredients available to her, ending up among the British expatriate community in Cairo and working as a reference librarian for the Ministry of Information. While in Cairo she married a Lieutenant-Colonel in the British Army, Tony David, and then went to India to assume (an unhappy) life as a memsahib in the last days of the British Raj. In 1946 she returned to England and began to write about her culinary experiences in the Mediterranean, to counteract her revulsion at food conditions in England. Steadily, she built herself a career in journalism as a food writer, working for publications such as *Harper’s Bazaar*, *Vogue*, the *Sunday Times*, and the *Spectator*. With the additional publication of several books, first on Mediterranean cuisine and then on English, David became hugely popular in the 1960s and 1970s among young middle-class metropolitans seeking to experiment with different approaches to food, including recovering older English traditions.

Throughout her life David pursued her own goals, caring little what others thought of her. She loved to drink, had extramarital affairs, loathed being a debutante, and supported individual women suffering from marital difficulties. David never identified as a feminist, not because she was somehow in thrall to the patriarchy (although she continued to style herself “Mrs. David” long after her divorce), but because she refused across the board to join groups and movements, preferring, sometimes at her own cost, to be a wanderer and a traveler, both physically and psychologically. She preferred to encounter the particularity and thus complexity thrown up by specific women’s situations, uninterested in “plans” to better the lives of some nebulous sisterhood (Chaney 350). Thus, she could say “I think that a woman’s position is terrible,” and yet never throw in her lot with “Women’s Lib” (Chaney 350). David’s silence on feminism, and hence on power as a problematic human motivation working against women, should not be read therefore as complicity; her focus was on food, and her conservative outlook—her preference for the particular and historically contingent and suspicion of ideology—permeated this too.

As the 1960s and 1970s wore on, David turned explicitly to English food traditions; her 1977 masterpiece and bestseller, *English Bread and Yeast Cookery* (hereafter *English Bread*), was a sustained attack on mechanical farming and the Chorleywood Bread Process that resulted in packaged sliced bread produced at a speed impossible if proving by hand—convenient for some, but tasteless and false for others. *English Bread* also tackled the ascendance of metric weights and measures in the early 1970s, promoted by the then Metrication Board and the broader drift of Britain into the European Economic Community (forerunner to the European Union). By taking issue with the “botheration” of metrication, David’s conservative stance became as explicit as it ever would (*English Bread* 233). Another “plan” of the French Revolution, metrication represented an “unnatural” attempt to determine how a range of diverse communities exchanged their goods and hence interacted with each other. David, echoing Burke, recognized that real people were more “haphazard,” especially in their “cooking measurements,” and could not realistically be treated as one homogenous group (234).

*English Bread* fits precisely into a canon of other twentieth-century writing by women rhetors of a conservative outlook of the kind outlined above, including Grigson and Ayrton, but also the earlier Florence White (1863-1940) and Dorothy Hartley (1893-1985), whose main project was to conserve practices and forms of knowledge (including weights and measures) that were at risk of being lost, and should be preserved because they were thought conducive to human happiness among distinct groups of people.^12^

*English Bread* also represented a marked shift in terms of the *kind* of food writing David practised. Her first book, *A Book of Mediterranean Food* consisted almost entirely of recipes, although written in an evocative, “literary” way that moved beyond straightforward instruction. Further books also contained recipes, but increasingly offered essays concerning the history of particular foods.

Generically speaking, David’s food writing sits firmly within the “gastronomic” end of the recipe-gastronomy spectrum devised by Stephen Mennell in 1985. Gastronomical writing has several characteristics, all of which are evident in David’s work: (1) a focus on cookery as an art form; (2) a heterogeneous writing style that includes literary allusions; (3) a transnational frame of reference; (4) consciousness of eating as a social affair; (5) a tendency towards self-indulgence; and (6) “a decidedly undomesticated approach toward pleasure” (McLean 4-5). By seeking to create works of gastronomic art rather than straightforward recipe books, David was, certainly in 1950, circumventing gendered expectations that women write recipes and men write gastronomy. It was only later, twenty-five years into David’s career, that Jane Grigson could say that there was also a tradition of women’s gastronomical writing in Britain, in which David played a crucial role in its development and greater visibility.

It is in this cultural moment that *An Omelette and a Glass of Wine* (hereafter *Omelette*) appeared, encouraged by David’s editor at Penguin, Jill Norman. *Omelette* synthesized David’s journalism from the 1950s through to the early 1980s and condensed David’s self-image as one concerned with writing about good food in the gastronomic tradition from a conservative angle that meditated on tradition in a non-rational way.

## David’s Pathos

The four essays on cookbooks in *Food, Feminisms, Rhetorics* rhetorically analyze how women rhetors produced and circulated modern recipe books, in which the recipe is considered “rational and highly reproducible” (Cognard-Black 33). Jennifer Cognard-Black shows how pathos resides “within the many modifications of the if/then recipe form […] where writers elicit historical, personal, communal, narrative, symbolic, and imagistic associations to appeal to their readers’ emotions” (34). Because David’s food writing is more gastronomic and refuses to rely solely upon recipes, its pathos is heightened, liberating her to meander through personal memories, historical narrative, evocations of foreign locations, as well as the food texts of other, primarily women, food writers of the past. It is my contention that David’s pathos is instructive for how it develops a conservative political culture in which food is presented as (1) a form of knowledge conducive to human happiness; and (2) a process by which traditions are formed in which such knowledge is conserved and passed to future generations where it is reverently received, adapted, and transmitted again.

For David, her journalism was an opportunity to persuade her readers to a way of engaging with food that would provide them with the know-how to experience moments of happiness and aesthetic pleasure. One of the ways she does this is by evoking scenes in which good and bad moments of food are personally experienced, such as in “Eating out in Provincial France 1965-1977,” published in 1980 in *Petits Propos.* David’s task in this essay is to describe a handful of “agreeable surprises,” moments that will inspire her readers to be more discriminating themselves about what they eat, for “[w]hat has suffered from the shrinkage is the quality of raw materials, of the cooking skills and also, I would say, the critical faculties of the customers” (rpt. in *An Omelette* 66). Firstly, David describes a humble restaurant in an inn “slap on the Bordeaux-Paris auto-route” (68). Nothing extraordinary here, except some excellent local sausages, but “[w]e left at daylight feeling, as travellers should, that we had been welcomed, comforted and cheered on our way. It was a good lesson in how the best hospitality is often to be found in the most unlikely of places” (68).

This little vignette serves as an *entrée* to the main course, a fabulous narration of dining at La Mère Brazier by the Col de la Luère, a few miles outside Lyon. As always with David, anything fancy is derided; that which is simple and good is cherished: “There was no showing off, no fireworks. The calm confidence, the certitude that all here would be as it should which one felt upon entering the establishment was somehow communicated to her customers by Madame Brazier herself” (rpt. in *An Omelette* 70-71). David develops pathos here by imaginatively relating a perfectly realistic scenario, almost prompting her readers to remember what this feels like. Everything in the restaurant seems “all of a piece” (71). Madame Brazier appears to focus on doing what she knows she can do well, in this case a *poularde de Bresse*, along with a salad of artichoke hearts and walnuts, “delicate and refreshing” (71). Even the dessert seems right, with a vanilla ice cream made *chez* Brazier: “I tried this once. It was a beauty” (71). David ends by summing up her experience of the food at Eugénie Brazier’s restaurant as “poetry,” thereby lifting her experience into the realm of aesthetic pleasure and reminding her readers how good food experiences communicate knowledge about pleasure in life (72).

By contrast, in “Confort anglaise, French fare,” first written in 1986 and which immediately follows “Eating out in Provincial France,” David describes an experience of eating which she did not enjoy, so that her readers are helped to discern the difference:

The grasping attitude, the general shabbiness, the brainless parsimony displayed by Madame, the dispirited and dispiriting service, the dreary bread, the absence of a house wine—always a bad sign in a shared restaurant—all added up to yet another of those dozens of unsolved Michelin mysteries of my past travels in France. (rpt. in *An Omelette* 80)

“Meanness” is singled out by David as the cardinal sin which restaurants are liable to commit because meanness signifies an overall violation of hospitality and the context in which happy eating experiences frequently occur (80).

Importantly, David uses pathos to heighten emotions in her readers in order to convince them of truths she knows cannot be argued logically. Even when she says that a certain cuisine, such as that from Provence, is “the rational, right and proper food for human beings to eat,” this is a statement arrived at by decidedly non-rational, imaginative means, in this case from a “vision of golden tiles on a round southern roof, or of some warm, stony, herb-scented hillside” which “will rise out of my kitchen pots with the smell of a piece of orange peel scenting a beef stew” (*French Provincial Cooking* 19).

The conservative philosopher Michael Oakeshott referred to the way in which a practical art such as cookery cannot be governed solely by rational principles, for “nobody supposes that the knowledge that belongs to the good cook is confined to what is or may be written down in the cookery book” (12-13). The kind of instinct that can sense if something is “right” is trained by immersing oneself aesthetically in food experiences—real or imaginative—and developing a feel for how others respond to good food. The problem David faced in the 1960s and 1970s was that she felt this instinct was in short supply: there simply were not enough opportunities for her readers to develop this sense for themselves, hence her rhetorical choice to express the benefits and pleasures of food in “lively terms” (12).

In addition to her non-rational method, what makes David’s pathos particularly conservative, however, is that she uses pathos to show how knowledge of good food is part and parcel of a living tradition, inviting historical reflection on the one hand and a feeling on the other for how food traditions of the past flourish in the present only by adapting them to present circumstances. In “Traditional Christmas Dishes,” published in *Vogue* in 1958, David addresses this challenge in relation to Christmas food. Quoting Philéas Gilbert, David states that “[c]ookery is as old as the world, but it must also remain, always, as modern as fashion” (rpt. in *An Omelette* 287). What David means by this is that when we try to cook according to old recipes—an admirable endeavor—we do so in ways that,

while based on the old ones, are modern in treatment. It is a system which works so long as the spirit of the recipes is preserved, for then we do get some sense of a continuing tradition into our cookery, avoiding the farcical effect produced by “traditional” recipes made up almost entirely of synthetic or substitute ingredients. (288)

Not only does David justify an approach to food that relies on the development of aesthetic instinct, she ties this sense to a conservative understanding of tradition and its role in political culture. Stemming from Edmund Burke, who famously said that “[a] state without the means of some change is without the means of its conservation,” twentieth-century British commentators on conservatism have similarly emphasized the importance of adaptation in the service of conserving habits, attitudes, practices, art forms, and much else to which ordinary people are often tied by bonds of affection (441-42). William B. Willcox noted that conservatives privilege “an attitude of mind which combines a love for the best of the past with a willingness to augment it cautiously with the best of the present” (716). David’s cousin, the Conservative MP and writer Quintin Hogg, disabused the notion that the wisdom of the past should remain fixed or unchanged, but rather viewed it as “a treasury to which it is the duty of each generation to make its characteristic contribution” (11), and Harvey Glickman reminded his readers in 1961 that “Reform and respect for tradition, but not traditionalism—not enslavement to the past—characterize the Tory belief” (125-26).

One of the most obvious ways in which David develops her conservative political culture of food tradition is by using pathos to bring tradition down to a human level. This works in two ways: (1) through personal anecdote of mentors in her past who helped her to a larger understanding of food and its role in human life, and (2) by narrating historical narratives of primarily women food writers of the past whose food traditions were at risk of being lost in a world in which their wisdom and knowledge were sorely needed.

Firstly, then, are David’s emotive portraits of her beloved mentor in *Omelette*: “South Wind through the Kitchen” for *Wine and Food*, 1964, and “Have It Your Way,” written for *Gourmet* in 1969. These are two portraits of the writer and raconteur Norman Douglas (1868-1952), whom David met in Antibes in 1940 and whose literary influence is felt in all that David subsequently wrote. In “Have It Your Way” David builds her readers’ emotional interest in what is, quite frankly, one of the most personal and private aspects of her life that profoundly shaped her commitment to food writing. A general maxim (“Always do as you please, and send everybody to Hell, and take the consequences”); a plea to end a relationship that was making her unhappy (“Stay here with me. Let him make do without you”); and a day in Norman’s company (“exhilarating; that little stroll rather less so”): these are woven together to create a poignant narrative which is cathartic for David and pathetic for her readers, who knew, with the benefit of hindsight, that these days Elizabeth had with Norman were numbered (120-23). And, as a mark of his influence on her, David explains that his philosophy of life, which reveals itself quite clearly in her writing on him, “emerged gradually, in the course of walks, sessions at the tavern, apropos a chance remark,” rather than through lectures (123). So too does her own treatment of his philosophy. Memories flicker into focus when she eats in a restaurant or visits a place in connection with him.

In “South Wind through the Kitchen” this is especially borne out by David’s visit to a lemon grove in the summer of 1952, months after Douglas died. Although she admits that she parted company with Douglas on his penchant for visiting graveyards, David nevertheless chooses to remember his memory by going to a grove they frequented together, and then evoking it for her readers. “It was so thick, that lemon grove,” she writes, “that it concealed from all but those who knew their Capri well the old Archbishops’ palace in which was housed yet another of those private taverns which appeared to materialize for Norman alone” (129). This is a scene of sensuality, almost a mirage that appears only to those who have their minds set on some beloved friend no longer physically present: “There, at a table outside the half-ruined house, a branch of piercingly aromatic lemons hanging within arm’s reach, a piece of bread and a bottle of the proprietor’s olive oil in front of me, a glass of wine in my hand, Norman was speaking” (129).

Most of David’s portraits of other food writers, however, were of women who wrote in the eighteenth and nineteenth centuries—women food writers who were also inspirational to Jane Grigson and Elisabeth Ayrton. For example, in “Welsh Doubles,” published in *Wine and Food* in 1965, David wrote of the advances made by Lady Llanover (1802-1896) of Llanover Estate, near Abergavenny in South Wales, whose *Good Cookery* of 1867 aimed to recover Welsh food traditions at risk of being lost by the march of Victorian progress. As Lady Llanover wrote in her introduction to this book: “My aim […] has been to preserve or restore all the *good* old habits of my country” (qtd. in 293). Music to her ears, David singles out the ways in which Lady Llanover contributed to the longevity of Welsh culture, often threatened by the dogma of progress: “In her repudiation of the marvels of Victorian progress and the products of what she called ‘mechanical talent’,” writes David, “she was also reactionary. Or was she a visionary? To those of us who today yearn increasingly for authenticity and natural food, she appears sometimes to be writing of the 1960s rather than the 1860s” (296). David’s treatment of Lady Llanover was actually *re*visionary; the scarcity of copies of *Good Cookery* in the 1960s symbolized Lady Llanover’s obscurity, yet David recasts this food writer as a visionary rather than a reactionary, who was brave enough to challenge orthodoxies that were also challenged by conservatives of the time who were concerned that liberal “progress” might uproot and displace in the name of advancing the lot of “universal mankind.” David, following Lady Llanover’s example, focuses on the particular, and how the particularity of food traditions can become a process in which knowledge about good food is preserved, transmitted, and adapted by those in new times. As the conservative philosopher Roger Scruton has noted:

In discussing tradition, we are not discussing arbitrary rules and conventions. We are discussing *answers* that have been discovered to enduring *questions*. These answers are tacit, shared, embodied in social practices and inarticulate expectations. Those who adopt them are not necessarily able to explain them, still less justify them. (*Green Philosophy* 220)

It is no accident then that David employs pathos in ways that align with her non-rational understanding of tradition as that which concerns knowledge of good things that are “tacit, shared, embodied in social practices” such as cookery.

By combining her historical research with the recovery of women food writers of the past, David also develops a conservative political culture in which, to echo Burke, culture itself is seen as a mutual task between different generations, which therefore requires specific emotions directed towards those who are no longer with us. In his *Green Philosophy*, Scruton has provided a perceptive and sensitive exploration of how emotion can mediate bonds between the dead, the living, and the unborn: “The time for which we yearn and to which we gravitate is one that stretches beyond this moment, this person and this life. It is a time in which the dead and unborn are also present” (234). Scruton is here drawing on Burke’s realization that because many of the things humans seek cannot be obtained in one generation only, “it becomes a partnership not only between those who are living, but between those who are living, those who are dead, and those who are to be born” (509). This sentiment is at the root of the traditional conservative insistence on incremental change and adaptation to the new, and it is a language of mutual aid riveted on an interplay of affect, imagination, and reverence rather than the negotiation of power.

David’s portraits of historical food writers, and her personal reminiscences of others who helped her to a larger understanding, are doing important rhetorical work in developing a conservative political culture of care. By homing in on the particular and eschewing the universal, David shows the importance of listening to the knowledge embodied in specific food traditions that are at risk of being lost within a dominant culture driven towards change and “the new.” While this could certainly be phrased in the language of “maneuvering amid systems of power,” David’s analysis of Lady Llanover, and David’s wider interest in individual women food writers of the past (as well as male ones), suggests that there are other organizing lenses through which to view food culture than power. In David’s conservative political culture, tradition is the process by which affection, imagination, and reverence are cultivated and which enables a caring partnership among the dead and the living, brought together over a common love of good things that need conserving.

## Conclusion

Elizabeth David’s food writing constitutes an important intervention in the development of feminist approaches to rhetoric. I have tried to show that by using pathos to explore the benefits of food traditions in conserving knowledge about good food, David’s conservatism is capable of inspiring emotions among feminist rhetoricians other than “dismay.” I have also used the case of food writing to show how conservatism—especially of David’s kind—models a broader political culture that affords listening space to human motivations other than power, particularly emotions of care. Post-war British conservatism enabled people to pursue a range of life concerns, not least the desire to feel connected to a past which, in the case of food, was threatened by forces (such as the Chorleywood Bread Process) the benefits of which were not always entirely evident. Feminist rhetorical work on women’s food writing undertaken with a genuine “ethics of hope and care” offers a way of expanding the range of conservatisms studied when turning to “conservative women,” and the British angle demonstrates the scope and imagination at work in the kind of conservative political culture David was at the forefront of developing.

## Notes

1 I wish to thank the two *RR* reviewers—Lisa Mastrangelo and Abby Dubisar—for their generous and constructive feedback during the review process.

2 See Steve Jones and Ben Taylor, Janet Floyd, Nicola Humble, and Alice L. McLean for existing scholarly assessments of David.

3 See especially the essays in volumes edited by Lindal Buchanan and Kathleen J. Ryan and Jacqueline Jones Royster and Gesa E. Kirsch.

4 See especially work by Catherine Brekus, Leslie Dorrough Smith, Michelle M. Nickerson, and Ronnee Schreiber.

5 For example, see the edited volume by Clarisse Berthèzene and Julie Gottlieb.

6 See also work by Nicola Humble.

7 Most recently, see work by Emily Robinson.

8 For explicitly conservative (party-political) rhetoric, see particularly Andrew Crines, Tom Heppell, Michael Hill, and Richard Shorten.

9 See especially David Goodhart’s analysis of British Social Attitudes Surveys since 1983.

10 See work by Roger Scruton.

11 For full details see biographies by Lisa Chaney and Artemis Cooper.

12 In fiction, the rural novels of Flora Thompson during the Second World War produced a similar impact.
